# Supplementary material for: Barriers to exclusive breastfeeding practice among HIV-positive mothers in Tanzania. An exploratory qualitative study
Source: PLoS One. 2024 May 28;19(5):e0296593. doi: 10.1371/journal.pone.0296593 (PMC11132465; doi:10.1371/journal.pone.0296593)
Supplement: S1 File — (PDF) [file pone.0296593.s001.pdf]

Guiding questions for individual interviews (English)

Introduction: Thank you for consenting to take part in this interview. This study aims to investigate the obstacles faced by HIV-positive women in Dar Es Salaam regarding exclusive breastfeeding practices. You have been chosen from among other participants due to your having a child between 3-6 months and attending follow-up in this clinic. There are no incorrect responses in this interview—all answers are welcome, so please feel free to express your thoughts. It's important to note that this interview is expected to last no more than 45 minutes and will be recorded. The recording captures nuanced details that may be challenging to document and facilitate easier information analysis at a later stage.

1. As an individual, what challenges have you encountered or did you face during the practice of Exclusive Breastfeeding (EBF) for your index child?
2. What challenges are you facing or have you encountered within your household during the practice of Exclusive Breastfeeding (EBF) for your index child?
3. What challenges are you currently facing or have you encountered within your community during the practice of Exclusive Breastfeeding (EBF) for your index child?

To conclude the interview: Is there anything else you want to add or share regarding our covered topics?

Thank you.

Guiding questions for individual interviews (Swahili)

Utangulizi: Asante kwa kukubali kushiriki katika mahojiano haya. Utafiti huu unalenga kuchunguza vikwazo vinavyokutana na wanawake wenye HIV hapa Dar Es Salaam kuhusiana na lishe ya kunyonyesha pekee. Umechaguliwa kutoka kati ya washiriki wengine kutokana na kuwa na mtoto kati ya miezi 3-6 na unapata matibabu katika kliniki hii. Hakuna majibu yasiyo sahihi katika mahojiano haya—majibu yote yanakaribishwa, hivyo tafadhali jisikie huru kutoa mawazo yako. Ni muhimu kutambua kwamba mahojiano haya yanatarajiwa kuchukua si zaidi ya dakika 45

na yatarekodiwa. Kurekodi kunakamata maelezo madogo yanayoweza kuwa magumu kuandikwa na hufanikisha uchambuzi rahisi wa habari baadaye.

1. Kama mtu binafsi, ni changamoto zipi umekutana nazo au umekumbana nazo wakati wa lishe ya Kunyonyesha Pekee (EBF) kwa mtoto wako?
2. Ni changamoto zipi unazokutana nazo au umekumbana nazo ndani ya nyumba yako wakati wa lishe ya Kunyonyesha Pekee (EBF) kwa mtoto wako?
3. Ni changamoto zipi unazokutana nazo sasa au umekumbana nazo ndani ya jamii yako wakati wa lishe ya Kunyonyesha Pekee (EBF) kwa mtoto wako?

Kuhitimisha mahojiano: Je, kuna kitu kingine ungependa kuongeza au kushiriki kuhusu mada tulizojadili?

Asante.

In-depth interview guide for Health Care Providers and Community Health Workers (English)

Introduction

Thank you sincerely for agreeing to take part in this interview. The objective of this study is to delve into the challenges confronted by HIV-positive women in Dar Es Salaam concerning exclusive breastfeeding practices. You have been chosen among other service providers due to your expertise in Exclusive Breastfeeding (EBF) practice and Prevention of Mother-to-Child Transmission (PMTCT) services. In this interview, there are no incorrect answers—all responses are welcomed, so please feel at ease to express your thoughts. It's important to note that this interview is anticipated to last no more than 45 minutes and will be recorded. The recording is designed to capture details that may be challenging to transcribe and facilitates the subsequent information analysis.

1. What challenges have your clients reported as individuals, either currently facing or having faced, during the practice of Exclusive Breastfeeding (EBF) for their index child?
2. What challenges have your clients reported or did they report facing within their households during the practice of Exclusive Breastfeeding (EBF) for their index child?

3. What challenges have your clients reported facing or having faced within their community during the practice of Exclusive Breastfeeding (EBF) for their index child?

To conclude the interview: Is there anything else you would like to add or share regarding our covered topics?

Thank you.

In-depth interview guide for health care providers and community health workers (Swahili)

Utangulizi: Asante sana kwa kukubali kushiriki katika mahojiano haya. Lengo la utafiti huu ni kuchunguza changamoto zinazokutana na wanawake wenye maambukizi ya VVU jijini Dar es Salaam kuhusu lishe ya kunyonyesha pekee. Umekuwa mmoja wa washiriki kutokana na utaalamu wako katika lishe ya Kunyonyesha Pekee (EBF) na huduma za Kupunguza Maambukizi kutoka kwa Mama kwenda kwa Mtoto (PMTCT). Katika mahojiano haya, hakuna majibu yasiyo sahihi—majibu yote yanakaribishwa, hivyo tafadhali jisikie huru kueleza mawazo yako. Ni muhimu kufahamu kwamba mahojiano haya yanatarajiwa kuchukua si zaidi ya dakika 45 na yatarekodiwa. Kurekodi kunalenga kukamata maelezo madogomadogo ambayo yanaweza kuwa magumu kuhakiki na kusaidia uchambuzi rahisi wa habari baadaye.

1. Ni changamoto zipi wateja wako wamekua wakikumbana nazo binafsi au umekumbana nazo wakati wa lishe ya Kunyonyesha Pekee (EBF) kwa mtoto wao wa kwanza?
2. Ni changamoto zipi wateja wako wamekua wakikumbana nazo kwenye makazi yao wakati wa lishe ya Kunyonyesha Pekee (EBF) kwa mtoto wao wa kwanza?
3. Ni changamoto zipi wateja wako wamekua wakikumbana nazo kwenye jamii kwa ujumla wakati wa lishe ya Kunyonyesha Pekee (EBF) kwa mtoto wao wa kwanza?

Kumaliza mahojiano: Je, kuna kitu kingine ungependa kuongeza au kushirikisha kuhusu mada tulizojadili?

Asante.
