# Supplementary material for: Barriers to exclusive breastfeeding practice among HIV-positive mothers in Tanzania. An exploratory qualitative study
Source: PLoS One. 2024 May 28;19(5):e0296593. doi: 10.1371/journal.pone.0296593 (PMC11132465; doi:10.1371/journal.pone.0296593)
Supplement: Attachment — Submitted filename: Response to Reviewers.docx [file pone.0296593.s002.docx]

**Kahsu Gebrekidan, PHD**

Academic Editor

PLOS ONE

Submission Date: February 22nd, 2024

Dear Dr. **Kahsu Gebrekidan**

Thank you for inviting us to submit a revised draft of our manuscript entitled " Infant and Young Child Feeding in the Context of HIV: An Exploration of Barriers in Exclusive Breastfeeding Practice in Dar es Salaam, Tanzania " to PLOS ONE. We also appreciate the time and effort you and each reviewer have dedicated to providing insightful feedback on ways to strengthen our paper. Thus, it is with great pleasure that we resubmit our article for further consideration. We have incorporated changes that reflect the detailed suggestions you have graciously provided. We hope our edits and responses below satisfactorily address all the issues and concerns you and the reviewers have noted.

To facilitate your review of our revisions, the following is a point-by-point response to the questions and comments in your letter dated Jan 8, 2024

|  | **REVIEWER 1** | |
| --- | --- | --- |
|  | **TITLE** | |
| 1. | The entire manuscript discusses about exclusive breast feeding which is one fore of IYCF. So, the current title is too broad. Limit it to exclusive breast-feeding practices in HIV positive women. And a clear title should include the problem, the subjects/study participants and the place. Consider this in your title too | Thank you for providing these insights, title has been modified to include problem, study participants and place while narrowed to specific infant feeding method (Exclusive breastfeeding) (page 1, lines 4-5), |
|  | **Material and methods** |  |
| 2. | 1. Data collection Description of who conducted the interview with the different participant groups and the interviews skill of collecting interview data (qualitative research) and what tools (audio recorder) were used to capture the interview with participants will be important to get clarity about your data collection. Include this information. How did you do verbatim transaction of each interview from field notes? | We have incorporated your comments by adding who conducted interview and relevant experience and skills of collecting interview data (page 5 lines 184-191) name of the tool used to capture the interview (page 5 line 193) and verbatim transcription of the interview (page 5 lines 202-204) |
| 3. | You have collected socio-demographic data to ensure participants from different socio demographic groups are represented in the interviews, which is good. If so, present the summary of participants characteristics in table. | Thank you for your suggestion. We have added a new Table 1 (6-7) which outlines Socio-demographic findings of the study participants |
|  | **Results** |  |
| 4. | 1. Treatment of different views Do all participant groups have similar view in each theme and sub theme described? If there are deviants, their view should be reflected. | In addressing the treatment of different views among participant groups within each theme and subtheme, it's important to acknowledge the diversity of the study population and the variability in perspectives that may arise from individual experiences and circumstances. While efforts were made to capture a comprehensive range of views, it's recognized that not all participant groups may have shared identical perspectives within each theme and subtheme. Deviant views or differing perspectives were considered and incorporated where relevant, ensuring a nuanced understanding of the barriers to exclusive breastfeeding across diverse contexts. The choice to present the most representative or predominant views within each subtheme was made to provide clarity and focus while still acknowledging the existence of alternative viewpoints within the study population.  Top of Form  Bottom of Form |
| 5. | 2. Triangulation How is the data from health professionals triangulated with finding from HIV positive women? Can you explain a bit further? | You have raised an important question, the interviewed healthcare providers were grouped in to two: Hospital based healthcare providers and Community based healthcare (CHW) provider/workers. Due to their different working environment and approach to their client hence different viewpoints to bring about triangulation of sources. |
|  | **REVIEWER 2** | |
|  | **Title** | |
| 1. | The title is fantastic and has brought some important findings. However, the study populations should be mentioned in the title. | Thank you for your suggestion, title has been modified to include study participants.  (page 1 lines 4-5) |
|  | **Abstract** |  |
| 2. | In abstract part, the methods part should contain study design, population, sample, sampling method, data collection, and type of data, tool, data analysis, and the way you interpreted. Please avoid traditional narration since qualitative research is “live” research. In my opinion, it is better to show the clear direction of exploring data from each source chronologically so that readers also understand where the insight information is documented and the method of exploration. | This is an interesting perspective, we have modified the methods part in abstract section ensuring clarity of study design, population, sample, sampling method, data collection, and type of data, tool, data analysis, and interpretation . (page 2 lines 54-61) Thank you for emphasizing the importance of clear direction and transparency in qualitative research reporting. |
| 3. | In abstract part: findings, you wrote, “The study identified various barriers to exclusive breastfeeding, encompassing individual factors." Please avoid introducing results to the word economy abstract; rather, just write the main findings and their link with study populations. Make the reader feel motivated to say the findings are live. | We agree with your assessment, we have modified the findings part of abstract to remain with main findings and their link with study population (page 2, lines 62-74) |
| 4. | In abstract part, Conclusion and Recommendations, I haven’t seen what you wrote here, in the findings. Theoretically, recommendations should emanate from results. | We agree with your assessment with modification that has been done to Conclusion and Recommendations with linkage to results we hope it’s better now. (page 2 lines 75-82) |
|  | **Introduction** |  |
| 5. | Introduction: Well written. However, since this is a qualitative study, you should analyse the individual, community, and health care-related factors beyond the numbers. Otherwise, it is fantastic. | Thank you for the complement and providing this insight. |
|  | **Methodology** |  |
| 6. | Please use the guidelines of the consolidated checklist for reporting in-depth interviews and focus group discussions in qualitative research. In my opinion, the current document lacks flow, especially in following the standard of qualitative research. The methodology needs a detailed description. Otherwise, well done! | We agree with you, we have used COnsolidated criteria for REporting Qualitative research (COREQ) throughout methodology for standard qualitative research and we think these changes are now better. (page 3-4, lines 119-199) |
| 7. | In Methods: As you can see from your method, there is no specific design explicitly written here. As to me, there are a variety of designs to be used in a qualitative study; please indicate the appropriate ones. | We agree that study design was not clearly reported, we have now modify (page 4, line 129-130) it to bring more clarity to the design used in this qualitative research. |
| 8. | In Methods: Data Collection and Tools, in-depth interviews and key informant interviews should be merged under the heading “Data collection methods and tools” and should be procedurally described. | Thank you for your suggestion, Data Collection and Tools, in-depth interviews and key informant interviews has been merged and procedurally described under the Data collection methods and tools. (page 5, lines 177-183) |
| 9. | In Methods: Data Management and Analysis Strategies is very well described. However, this part should need a detailed description of the “trustworthiness of qualitative research." Which is missed! | We agree with your assessment, Data Management and Analysis Strategies has been well revised to increase transparency of the strategies used hence to improve trustworthiness (page 5-6, lines 201-220) |
|  | **Results** |  |
| 10. | In result part, I found it very nice. However, conceptualize the whole concept of the emerging themes and anomalies in the beginning so that readers can be interested. | You have raised an important point; however, we believe that this has been done through the table to introduce themes and sub themes (page 8, line 237), and inclusion of the subthemes in the introduction part of the each theme (page 8, lines 239-243), (page 10, lines 326-330) and (page 12, lines 405-407). |
| 11. | Table 1 should be formatted and auto-fit to the window. Otherwise, it is fantastic. | Thank you for the complement, Table 1 has been formatted and auto-fit to the window (page 8, line 237) |
| 12. | This is a new view of the result or study; perhaps I am expecting from you to include the relationships between the variables obtained from your result in network or diagram form in order to build the idea. Since in qualitative research, all is data. | Thank you for the suggestion. I'll aim to include the relationships between variables from the results in network or diagram form in the next submission to enhance the visualization of qualitative data. It has been challenging to achieve a clear construct, but I am actively working on refining it. |
|  | **Discussion** |  |
| 13. | In discussion part, it was well narrated and discussed. | Thank you so much. |
|  | **Limitation** |  |
| 14. | Putting honest limitations first is best. | We have incorporated your comment by putting honest limitation first (page 16, lines 574 – 575) |
|  | **References** |  |
| 15. | References: Please make sure that all the references are used in the document and updated. | References has been updated to ensure all have been used in the document. (page 17-23) |

Thank you for allowing us to strengthen our manuscript with your valuable comments and queries. We have worked hard to incorporate your feedback and hope these revisions persuade you to accept our submission.

Sincerely,

Goodluck Augustino Mosha

Corresponding Author

School of Public Health and Social Sciences

Muhimbili University of Health and Allied Sciences

9 United Nations Road, Upanga West 11103 Dar Es Salaam, Tanzania

goodluckaugustino@gmail.com

+255-712443312

Additional Contact [should the corresponding author not be available]

Amani Anaeli

School of Public Health and Social Sciences

Muhimbili University of Health and Allied Sciences

9 United Nations Road, Upanga West 11103 Dar Es Salaam, Tanzania

[amanianaeli@yahoo.co.uk](mailto:amanianaeli@yahoo.co.uk)

+255-71344372
